# Reactions Related to CAR-T Cell Therapy

**DOI:** 10.3389/fimmu.2021.663201

**Published:** 2021-04-28

**Authors:** Lele Miao, Zhengchao Zhang, Zhijian Ren, Yumin Li

**Affiliations:** ^1^ Department of General Surgery, Second Hospital of Lanzhou University, Lanzhou, China; ^2^ Key Laboratory of the Digestive System Tumors of Gansu Province, Second Hospital of Lanzhou University, Lanzhou, China

**Keywords:** chimeric antigen receptor T cell, immunotherapy, adverse reactions, mechanism, coping strategies

## Abstract

The application of chimeric antigen receptor (CAR) T-cell therapy as a tumor immunotherapy has received great interest in recent years. This therapeutic approach has been used to treat hematological malignancies solid tumors. However, it is associated with adverse reactions such as, cytokine release syndrome (CRS), immune effector cell-associated neurotoxicity syndrome (ICANS), off-target effects, anaphylaxis, infections associated with CAR-T-cell infusion (CTI), tumor lysis syndrome (TLS), B-cell dysplasia, hemophagocytic lymphohistiocytosis (HLH)/macrophage activation syndrome (MAS) and coagulation disorders. These adverse reactions can be life-threatening, and thus they should be identified early and treated effectively. In this paper, we review the adverse reactions associated with CAR-T cells, the mechanisms driving such adverse reactions, and strategies to subvert them. This review will provide important reference data to guide clinical application of CAR-T cell therapy.

## Introduction

Several studies have explored various methods for treatment of malignant tumors. In 2008, American immunologists James P Alison and Japanese immunologists Tasuku Honjo won the 2018 Nobel Prize in physiology or medicine for their contributions to the field of tumor immunity. The findings of the study provide basis for development of novel treatment methods for malignant tumors, and indicate that tumor immunotherapy has a huge potential for treatment of various tumors. CAR-T cell therapy, a type of tumor immunotherapy has been widely explored over the past few years, and is widely used in treatment of malignant tumors. CARs can target any cell surface molecules. CAR does not require antigen processing or human lymphocyte antigen (HLA) presentation unlike T cell receptor (TCR)-modified T cells. Therefore, it is broadly applicable to patient populations with different HLAs (1, 2).

Although advances in CAR-T cell therapy for treatment of hematological malignancies have been reported, it is associated with severe adverse reactions some of which are life-threatening. This paper reviews adverse reactions that occur during treatment with CAR-T cells, their formation mechanisms, and strategies for alleviating them. The findings of this study provide a basis for clinicians to improve management of adverse reactions related to CAR-T cell therapy.

## Adverse Reactions Related to CAR-T Cell Therapy

### Cytokine Release Syndrome (CRS)

CRS, also known as “cytokine storm”, refers to the systemic inflammatory response syndrome caused by infection or administration of various drugs. CRS was first reported in 1990 in clinical trials exploring monoclonal antibody OKT3 as an immunosuppressant for kidney transplant patients (3). Advances in development of CAR-T cell therapy in recent years, has resulted in many studies exploring CRS which is the most common adverse reaction for this therapy. Studies report that incidence of CRS in CAR-T cell experiments targeting CD19 and BCMA is significantly high (**Table 1**). Previous meta-analysis reported that the incidence of CRS in patients with hematological malignancies receiving CAR-T cell therapy is approximately 55.3% (16), and the incidence of severe cytokine release syndrome (sCRS) is approximately 18.5% (17). In addition, use of immune-targeted drugs such as Nivolumab (18, 19) and Brentuximab Vedotin (20) is associated with severe CRS. In 2018, the American Society for Blood and Marrow Transplantation (ASBMT) stated that if after receiving any immunotherapy, the patient’s endogenous or induced immune effector cells are activated in large numbers, then symptoms for the resulting superphysiological response must include fever and may also include hypotension, capillary leakage (hypoxia), and end-organ dysfunction for the response to be referred as CRS (21). The new definition expands application of the term as it is not limited to CAR-T cell therapy. Mild clinical manifestations of CRS include fever (mainly the first symptom), fatigue, headache, joint pain, and myalgia. Notably, severe cases are characterized by hypotension and high fever. Further exacerbations may cause shock, vascular leakage, disseminated intravascular coagulation (DIC), and multiple organ dysfunction syndrome (MODS) (22).

**Table 1:** Summary of the incidence of CRS and ICANS in patients with hematological malignant tumors (partial data).

Time	Disease	CAR-T Cell Therapy	Phase	Case	Age (years)	Any Grade		Gade 3/4		Trial registration
						CRS	ICANS	CRS	ICANS	
2019	relapsed / refractory MM	BCMA-CAR-T cells	I	33	37~75	25 (76%)	14 (42%)	2 (6%)	1 (3%)	NCT02658929 (4)
2019	relapsed/refractory B-ALL	CD19-CAR-T cells	I	25	1~22.5	20 (80%)	18 (72%)	4 (16%)	7 (28%)	NCT01860937 (5)
2019	relapsed / refractory MM	BCMA-CAR-T cells	I	25	44~75	22 (88%)	8 (32%)	8 (32%)	3 (12%)	NCT02546167 (6)
2019	relapsed / refractory MM	BCMA-CAR-T cells	I	17	35~73	17 (100%)	**---**	6 (35%)	**---**	NCT02435849 (7)
2019	relapsed/refractory B-ALL	CD19-CAR-T cells	I/II	53	0~65	53 (100%)	8 (15%)	19 (36%)	**---**	NCT02965092 (8)
2019	relapsed / refractory MM	BCMA-CAR-T cells + CD19-CAR-T cells	II	21	49.5~61	16 (91)	2 (10%)	1 (5%)	**---**	ChiCTR-OIC-17011272 (9)
2019	relapsed or refractory diffuse large B-cell lymphomas	CD19-CAR-T cells	IIa	111	22~76	64 (58%)	23 (21%)	24 (22%)	13 (12%)	NCT02445248 (10)
2020	relapsed/refractory B-ALL	CD19-CAR-T cells	I	23	10~67	18 (78.3%)	3 (13%)	5 (21.7%)	1 (4)	ChiCTR-ONN-16009862, ChiCTR-1800019622 (11)
2020	relapsed or refractory large B-cell lymphomas	CD19-CAR-T cells	I	269	54~70	113 (42%)	80 (30%)	6 (2%)	27 (10%)	NCT02631044 (12)
2020	relapsed / refractory mantle-cell lymphoma	CD19-CAR-T cells	II	68	38~79	62 (91%)	43 (63%)	10 (15%)	21 (31%)	NCT02601313 (13)
2021	relapsed / refractory MM	BCMA-CAR-T cells	II	128	33~78	107 (84%)	23 (18%)	7 (5%)	4 (3%)	NCT03361748 (14)
2021	relapsed / refractory DLBCL	BCMA-CAR-T cells	I	31	24~82	23 (74%)	14 (45%)	0	10 (32%)	**---** (15)

MM, multiple myeloma; B-ALL, B-cell acute lymphoblastic leukemia; DLBCL, diffuse large B‐cell lymphoma; BCMA, B-cell maturation antigen.

—: Relevant data are not mentioned in the experiment.

**Figure 1:**
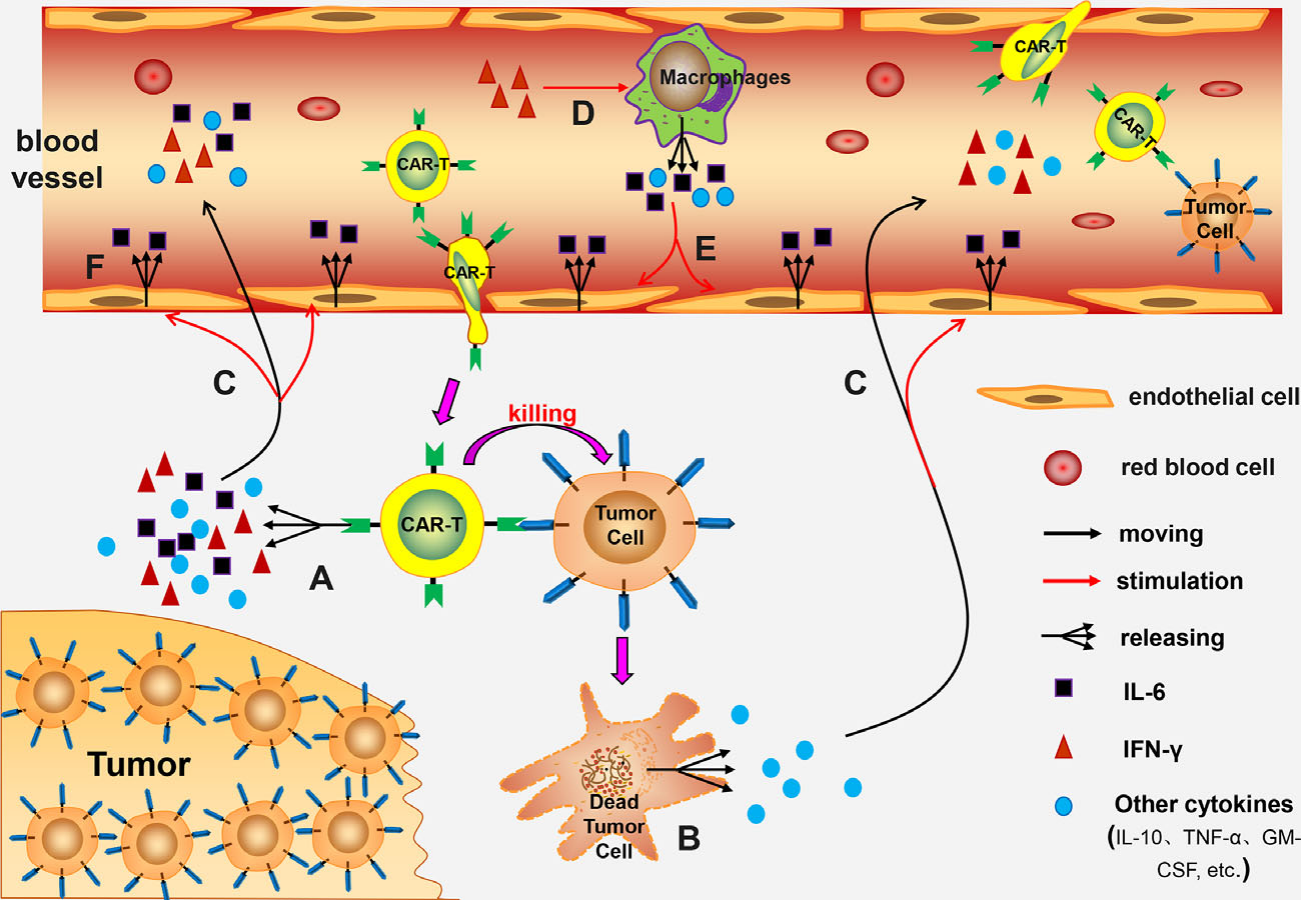
The mechanism of CRS. **(A)** Activated CAR-T cells release numerous cytokines; **(B)** The lysed tumor cells release a large number of cytokines; **(C)** These cytokines enter the blood circulation and activate endothelial cells; **(D)** IFN-γ further induces macrophages activation; **(E)** The activated macrophages release many cytokines into the blood circulation which activate endothelial cells; **(F)** The activated endothelial cells release large amounts of IL-6, forming a vicious circle.

Pathogenesis of CRS associated with CAR-T cell therapy has not been fully explored. Previous experimental studies report that pathogenesis may be linked to the following mechanisms: (a) CAR-T cells release numerous cytokines after being activated, such as IL-6, IL-10, TNF-α, GM-CSF, and IFN-γ. Out of these cells levels of IL-6 are significantly higher compared with the levels of other cytokines. These cytokines thus induce CRS (23, 24). (b) Lysed tumor cells release a high amounts of cytokines, such as TNF-α (22). (c) IFN-γ induces activation of immune cells, especially macrophages. Activated macrophages release several cytokines, such as IL-6, IL-1, IL-10, TNF-α, and NO (22, 24–26). Norelli et al. performed a study using mouse animal models and reported that IL-1 and IL-6 derived from monocytes are cytokines necessary for production of CRS and neurotoxicity (26). (d) IL-6 can induce a strong immune response and play a key role in production of CRS (26). (e) Release of high amounts of cytokines, such as IL-6, TNF-α, and IFN-γ, can induce activation of endothelial cells. The activated endothelial cells play a significant role in the pathological process of CRS. Activated endothelial cells secrete large quantities of IL-6, resulting in a vicious circle (27, 28). (f) Incidence and severity of CRS are positively correlated with the patient’s tumor burden (29, 30). This can be attributed to activation of several CAR-T cells or destruction of high number of tumor cells being in the body (**Figure 1**).

Clinically, different treatment plans are used for treatment of CRS based on the CRS grade. Symptomatic treatment is adopted for patients with grade 1 CRS; supportive and symptomatic treatment are adopted for patients with grade 2 CRS, and patients who are elderly or have serious complications should receive immunosuppressive therapy. Supportive treatment and immunosuppressants are adopted for patients with grade 3 and 4 CRS (31). Most commonly used immunosuppressants include Tocilizumab and glucocorticoids. The latest ASTCT consensus recommends use of Tocilizumab for CRS ≥2. The dosage regimen is as follows: patients with body weight ≥ 30kg are administered with 8mg/kg; patients with body weight < 30kg with 12mg/kg and the maximum dose should be ≤ 800mg/dose (32). Furthermore, a slow intravenous administration (>1h) should be adopted and the administration frequency should be ≤ 4 times. Tocilizumab blocks membrane-bound and soluble IL-6 receptors, and prevents IL-6 from binding to its receptors through competitive inhibition. It then neutralizes the activity of IL-6 by classic signaling, trans-signaling and trans-presentation (33, 34). Therefore, tocilizumab is an antagonist of IL-6 receptor, and can block the vicious circle of IL-6 in CRS through the above-mentioned mechanisms. Tocilizumab rapidly relieves the clinical symptoms of CRS without affecting proliferation and anti-tumor activity of CAR-T cells (29, 35). It has been approved by the United States Food and Drug Administration (FDA) as a first-line drug for management of CRS caused by CAR-T cell therapy (36). If the symptoms cannot be alleviated or relief is not evident, tocilizumab should be replaced with glucocorticoids or a combinatory therapy of glucocorticoids and tocilizumab used. The high immunosuppressive effect of glucocorticoids plays an important role in treatment of ≥3 grade CRS. Glucocorticoid administration schedule is as follows: patients with grade 3 CRS are administered with methylprednisolone (2mg/kg/day) or dexamethasone (10mg/6h) whereas patients with grade 4 CRS are given methylprednisolone (1000mg/day for 3 consecutive days) (37, 38): Glucocorticoids affect proliferation and activity of CAR-T cells in the body (39, 40). However, several studies have reported contradicting results recently. Some studies report that glucocorticoids do not affect activity and efficacy of CAR-T cells (41, 42). However, glucocorticoids have significant immunosuppressive effect, therefore, the dosage and the treatment course of glucocorticoids should be designed carefully. Sachdeva et al. (43) reported that granulocyte-macrophage colony-stimulating factor (GM-CSF) is a key CRS-promoting protein. In addition, the findings showed that the incidence rate of CRS in GM-CSF-knockout CAR-T cells is significantly lower compared with the rate of CRS in GM-CSF-intact CAR-T cells. These findings imply that inhibition of GM-CSF can prevent occurrence of CRS. Furthermore, therapeutic plasma exchange (TPE) and hemofiltration are potential therapies for some patients with severe CRS (44, 45).

Currently, diagnosis of CRS mainly depends on clinical manifestations of patients. Cytokines or other biomarkers related to occurrence and development of CRS are screened, and targeted intervention and treatment is administered on time based on changes in levels of these biomarkers, to effectively prevent, block and treat CRS. However, it is challenging to find key cytokines of CRS and their associated treatment time points. This is mainly because CRS is caused by a variety of cytokines and multiple factors. Furthermore, the onset time of CRS is different in different patients. Some patients may develop CRS on the first or second day after receiving CAR-T cells, and some patients may develop CRS several days later or even later, therefore the timing of detecting cytokine changes is not definite. In addition, several types of related cytokines are present during the onset of CRS, and it is expensive to detect CRS-related cytokines at regular intervals (a few hours or every day). The optimum time point for anti-cytokine therapy for CRS is still being investigated. Freyer et al. (46) reports that detection of CRS is done through clinical diagnosis, and inflammatory cytokine profiles are useful in confirmatory tests; however, these data are not used to determine the grade or treatment of CRS. C-reactive protein (CRP) and ferritin are correlated with the severity of CRS and can be used as biomarkers to determine the grade of CRS (27, 47). In addition, Hay et al. (27) reported that monocyte chemoattractant protein-1 (MCP-1) has higher specificity and sensitivity in predicting and diagnosing grade 4 CRS. The level of MCP-1 in blood increased (>1343.5pg/mL) accompanied by fever (≥ 38.9C) within 36 hours after receiving CAR-T cells. Sensitivity and specificity of predicting grade 4 CRS were 100% and 95%, respectively.

### Immune Effector Cell-Associated Neurotoxicity Syndrome (ICANS)

ICANS also known as neurotoxicity, is the second most frequent adverse event of CAR-T cell therapy. It can occur simultaneously with or after presentation of CRS. ICANS is activation or participation of T cells (autologous or exogenous) and/or other immune cells after receiving any immunotherapy, resulting in neurotoxic symptoms (21). The term of CAR-T-cell-related encephalopathy syndrome (CRES) is relatively limited. CRES only refers to neurotoxic symptoms caused by CAR-T cell therapy, therefore the term ICANS is commonly used. CAR-T cell experiments targeting CD19 and BCMA, report significantly high incidence of ICANS (**Table 1**). A previous meta-analysis reported that the incidence of ICANS in patients with hematological malignancies receiving CAR-T cell therapy was approximately 37.2% (16), whereas a different meta-analysis reported that the incidence of ICANS was approximately 21.7% (17). The main manifestation of CRES is toxic encephalopathy. Early symptoms include reduced attention, and language, and writing disorders. Other symptoms and signs include confusion, lethargy, and tremor. In severe cases, seizures, motor weakness, elevated intracranial pressure (ICP), and cerebral edema are reported (48–51).

Pathogenesis of ICANS has not been fully explored. Studies report that ICANS pathogenesis may be related to the following factors: (a) Levels of IL-1, IL-6, IL-15, TNF-α, and IFN-γ in blood are elevated, and are positively correlated with severity of ICANS (31, 38, 52, 53). These cytokines facilitate development and progression of CRES; (b) Activation of endothelial cells of the central nervous system (CNS) results in an increase in permeability of blood-brain barrier (BBB), which allows cytokines in the blood to enter the cerebrospinal fluid (CSF) and promotes development of ICANS (54, 55). The protein content of cerebrospinal fluid in CRES patients is high, implying that the BBB is destroyed (38). (c) CAR-T cells can enter the CSF and damage the CNS (52, 56). Studies report that the number of CAR-T cells in CSF of ICANS patients is significantly higher compared with that of patients without CRES (29, 52, 56). (d) Incidence of ICANS is positively correlated with the tumor burden and severity of CRS (31, 53). Early prevention and intervention of CRS may reduce occurrence of ICANS to a certain extent. Although tocilizumab can treat CRS, it has little or no effect on ICANS, primarily because it cannot penetrate BBB (29, 31).

The therapeutic regimens of ICANS include (38) fasting and intake of water, nutritional support treatment, and improved neurological examination (i.e., electroencephalogram, 30 minutes per day)for grade 1 ICANS whereas tocilizumab (8mg/kg, intravenous administration> 1 hour) or siltuximab (11mg/kg, intravenous administration> 1 hour) can be administered for grade 1 ICANS with CRS. In addition, tocilizumab (8mg/kg, intravenous administration>1 hour) or siltuximab (11mg/kg, intravenous administration>1 hour) is administered for grade 2 ICANS. If the above drugs are ineffective or poorly effective or a comorbidity of ICANS with CRS is reported, glucocorticoids (dexamethasone 10mg/kg/6h or methylprednisolone 1mg/kg/12h) should be administered. For grade 3 ICANS, it is recommended to transfer patients to ICU for further treatment and administration of glucocorticoid therapy (the dose is the same as above, until the patient’s condition improves to grade 1 ICANS, and then the dose is gradually reduced). Patients with grade 4 ICANS are given high-dose glucocorticoid (methylprednisolone 1g/day for 3 days, then the dose is gradually reduced; the whole course of treatment is 9 days). Further, ICANS patients with CRES-related seizures are treated with glucocorticoids combined with levetiracetam (500-1000mg/12h) whereas ICANS patients with raised intracranial pressure should be treated with glucocorticoids combined with acetazolamide. ICANS patients with cerebral edema should receive high-dose glucocorticoids, and hyperventilation and hypertonic therapy (for example, mannitol: initial dose 0.5-1g/kg/6h, Maintenance dose 0.25–1 g/kg/6 h) should be administered concurrently. Unlike tocilizumab, siltuximab is a monoclonal antibody binding to IL-6. It has a high affinity to IL-6 and can prevent binding of IL-6 to its receptor. Gust et al. speculated that tocilizumab may increase the level of IL-6 in CSF and aggravate neurotoxicity; however, administration of siltuximab (IL-6 antagonist) does not increase the level of IL-6 in CSF; therefore, siltuximab can be used as the first choice drug for treatment of ICANS (55). In addition, Norelli et al. (26) reported that IL-1 and IL-6 derived from monocytes played an important role in occurrence and development of CRS and ICNAS in mouse models. Early use of anakinra (IL-1 receptor inhibitor) can effectively prevent CRS and ICANS.

Currently, diagnosis of ICANS mainly depends on clinical manifestations of patients. However, similar to CRS, it is challenging to find key cytokines associated with ICANS pathogenesis and the time points of treatment related to them. CRP and ferritin can be used in prediction and diagnosis of ICANS. Karschnia et al. (57) analyzed the acute-phase protein levels in serum of 25 patients who developed ICANS after CAR-T cell therapy. The findings showed increase in the levels of CRP and ferritin in most patients after receiving CAR-T cells. Notably, the CRP reached a maximum level before appearance of neurological symptoms. On the other hand, the level of ferritin peaked after the onset of neurological symptoms. Moreover, ferritin level of patients with high-grade ICANS reached a peak (4533 ± 930 ng/mL, normal value: 1646 ± 472 ng/mL) 2 days after onset of neurological symptoms.

### Off-Target Effects

For effective targeted therapy, tumor antigens to be targeted should only be expressed on tumor cells, and not expressed or expressed in very low on normal cells. These tumor antigens are known as tumor-specific antigens (TSAs). However, TSAs are few, and tumor-associated antigens (TAAs) are mainly used for targeted therapy [For example in digestive tumors, some TAAs are targeted by CAR-T cells (**Table 2**)]. CAR-T cells injected into the body kill tumor cells expressing the target antigens, and normal cells expressing the target antigens. This phenomenon is known as on-target-of-tissue effects. These effects sometimes can cause severe side effects and even death. Morgan et al. (89) developed CAR-T cells targeting ERBB2 (HER-2/neu) for treatment of cancer patients with ERBB2 overexpression. One patient with colon cancer that had metastasized to the lungs and liver received this treatment. Within 15 minutes of infusion with CAR-T cells, the patient developed respiratory distress. Chest X-rays showed pulmonary infiltration, and despite aggressive medical intervention, the patient died 5 days later. The researchers speculate that CAR-T cells entered the lungs after infusion. In this case, CAR-T cells targeted lung epithelial cells with low ERBB2 expression and a large number of cytokines were released, resulting in CRS. Use of TSAs to develop the corresponding CAR-T cells is an effective method to eliminate off-target effects. However, finding new TSAs is challenging and expensive. Therefore, studies should aim at increasing the specificity of CAR-T cells by optimizing the structure of CAR. Commonly used methods of increasing efficacy include synNotch receptor (90) and inhibitory CAR (iCAR) (91).

**Table 2:** Some TAAs targeted by CAR-T cells (taking gastrointestinal tumors as an example).

Associated Malignancy	Target Antigens	Co-stimulating domain	Generation of CAR-T	Authors	Reference
**esophageal cancer**	EphA2	4-1BB	2^nd^	Shi, et al.	(58)
**liver**	NKG2DL	4-1BB	2^nd^	Sun, et al.	(59)
**cancer**	GPC3	CD28	2^nd^	Wu, et al.	(60)
		CD28,4-1BB	3^rd^	Jiang, et al.	(61)
		CD28, ICOSL	3^rd^	Hu, et al.	(62)
		CD28	4^th^ (IL12)	Liu, et al.	(63)
		CD28,4-1BB	4^th^ (IL15,IL21)	Batra, et al.	(64)
	CD147	4-1BB	2^nd^	Zhang, et al.	(65)
	AFP	CD28	2^nd^	Liu, et al.	(66)
**gastric**	Trop2	CD28,4-1BB	3^rd^	Zhao, et al.	(67)
**cancer**	PD-L1	CD28,4-1BB	3^rd^	Zhao, et al.	(67)
	CLDN18.2	CD28 or 4-1BB	2^nd^	Jiang, et al.	(68)
	FOLR1	CD28	2^nd^	Kim, et al.	(69)
	HER2	4-1BB	2^nd^	Han, et al. ;Song, et al.	(70, 71)
	MSLN	CD28	2^nd^	LV, et al.	(72)
	NKG2DL	4-1BB	2^nd^	Tao, et al.	(73)
**pancreatic**	CEA	CD28	4^th^ (IL18)	Chmielewski, et al.	(74)
**cancer**		CD28	2^nd^	Chmielewski, et al.	(75)
	HER2	4-1BB	2^nd^	Raj, et al.	(76)
	FAP	4-1BB	2^nd^	Lo, et al.	(77)
	CD47	CD28	2^nd^	Golubovskaya, et al.	(78)
	tMUC1	CD28	2^nd^	Yazdanifar, et al.	(79)
	B7-H3	CD28 or 4-1BB	2^nd^	Du, et al.	(80)
	MSLN	4-1BB	2^rd^	Zhang, et al.	(81)
	PD-L1	CD28,4-1BB	3^rd^	Yang, et al.	(82)
**colorectal**	GUCY2C	CD28,4-1BB	3^rd^	Magee, et al.	(83, 84)
**cancer**	NKG2DL	CD28,4-1BB	3^rd^	Deng, et al.	(85)
	CEA	CD28	2^nd^	Zhang, et al.	(86)
	DCLK1	CD28	2^nd^	Sureban, et al.	(87)
	EpCAM	CD28,4-1BB	3^rd^	Zhang, et al.	(88)

Taking gastrointestinal tumors as an example, the above-mentioned commonly used CAR-T cell targets are almost all TAAs.

### Anaphylaxis

Most CAR-T cells currently used in clinical trials contain antigen-recognition domains derived from murine monoclonal antibody (mAb) (92), which may be the major cause of anaphylaxis. In a previous clinical trial (93), four patients received multiple injections of MSLN (mesothelin)-CAR-T cells, and one of them developed cardiorespiratory failure at the end of the third infusion. Analysis showed presence of human anti-mouse antibodies and elevated trypsin antibodies in the patient’s serum, implying that it was an IgE-mediated anaphylactic event. The adverse effects observed during the trial may have caused by isotype switching to IgE (meaning that the specificity of the antibody remains the same, but its effects change). In addition, inappropriate timing of treatment also contributes to this situation, including treatment intervals. In another study (94), the antigen-recognition region of CAR was designed to contain only one human heavy-chain variable domain without a light-chain region or a linker. These CARs showed reduced immunogenicity and significantly reduced the size of the CAR-binding domains compared with the traditional CARs. Notably, these CARs did not show decrease in proliferation and tumor-killing effects of these CAR-T cells. Two major strategies are used in reducing immunogenicity of CAR-T cells including: (a) use of complete human sequences rather than murine sequences when constructing CAR; (b) Simplifying the structure of CAR (94–97). For patients receiving CAR-T cell therapy, the most important thing is to closely monitor changes in the condition and treatment of anaphylaxis in time. The treatment can be suspended or even terminated in case the effects are alleviated or if no response is observed.

### Infections Associated With CAR-T-Cell Infusion (CTI)

Infections associated with CTI are relatively common in CAR-T cell therapy. In a clinical trial using CD19-CAR-T cells to treat relapsed B-cell acute lymphoblastic leukemia (B-ALL), about 42% of 53 patients developed infections during the first 30 days after CTI. The infections were mainly bacterial, with bloodstream infections (BSIs) showing the highest incidence (98). From 31st to 180th day after CTI, 31% of the 32 patient survivors in complete remission developed infections, mainly viral infection with respiratory viruses being more frequent.

**Figure 2:**
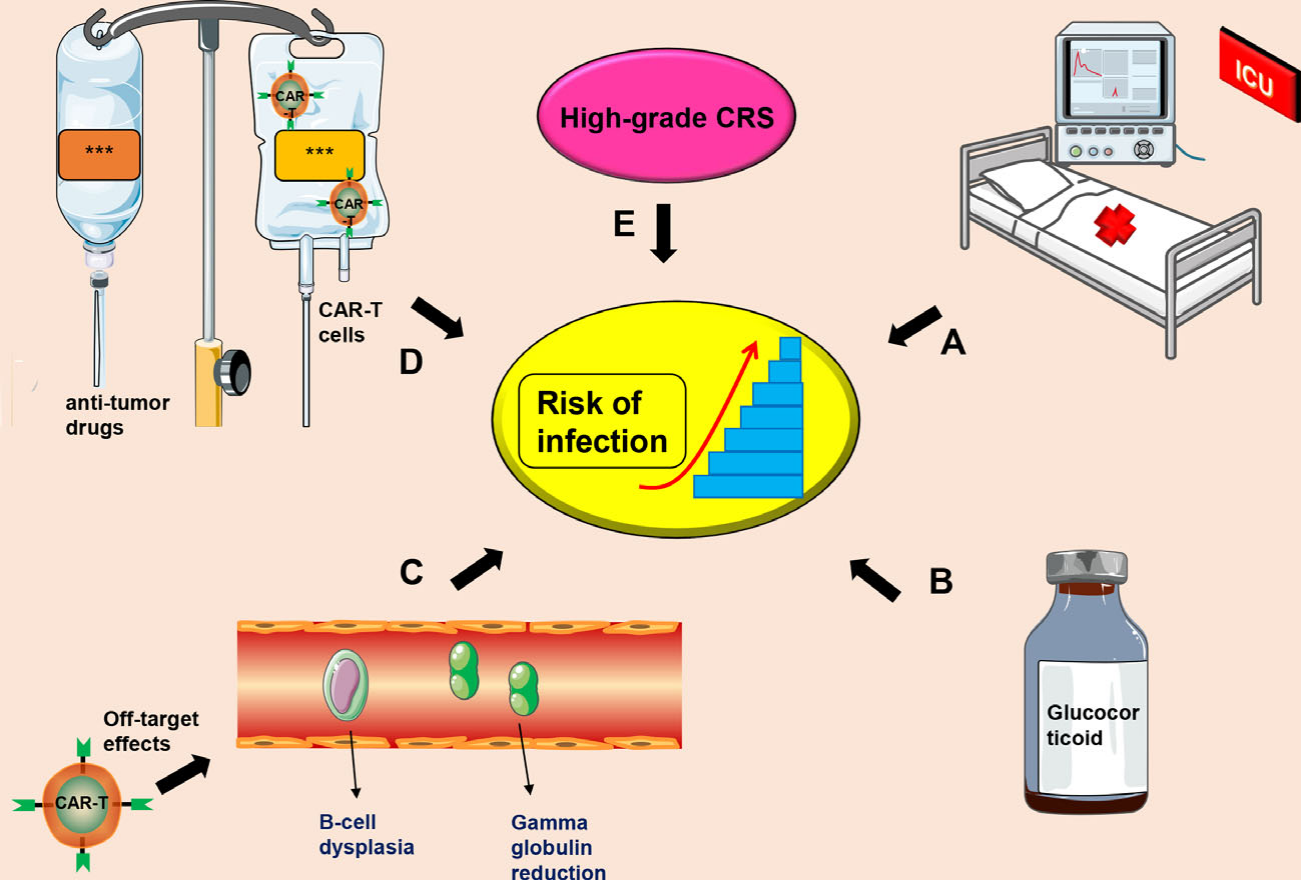
Factors that induce CIT. **(A)** Severe CRS patients receiving further treatment in the ICU, which increases the risk of infection; **(B)** Long-term administration of high-dose use of glucocorticoids; **(C)** Off-target effects of CAR-T cell therapy may cause B-cell dysplasia and hypogammaglobulinemia; **(D)** A combination of anti-tumor drugs and CAR-T cells; **(E)** High-grade CRS. This symbol *** only represents drug information or patient information.

Currently, the mechanism of CTI occurrence is unclear, and there is no unified treatment plan for prevention and treatment of CTI. The most commonly used CD19-CAR-T cell therapy and causes of infection after CTI may include (a) Severe CRS and/or CRES as a result of CAR-T cell therapy. These patients mostly undergo treatment in intensive care unit (ICU), which may increase the risk of nosocomial infections (31, 99); (b) Long-term and high-dose use of glucocorticoids for treatment of severe CRS and/or CRES can reduce the patient’s immunity (31, 38); (c) CD19-CAR-T cells can cause B-cell dysplasia and hypogammaglobulinemia, increasing risks of infection (29, 100, 101); (d) Patients receiving stronger anti-tumor drugs and high-dose CAR-T cells may have a higher risk of infection (102); (e) High-grade CRS is significantly positively correlated with risk of infection (98) (**Figure 2**).

The main prevention and treatment measures for CTI related infections include (a) Paying attention to protection and avoiding cross-infections; (b) Administration of antibiotics and immunoglobulins to prevent and treat infections (102); (c) Reduction of the duration for glucocorticoids administration; (d) Active treatment of CRS; (e) Reduction of the input dose of CAR-T cells. Notably, patients receiving CD19-CAR-T cells rarely develop lethal infections (98, 102).

### Tumor Lysis Syndrome (TLS)

A large number of tumor cells appear necrosis in a short time, resulting in the release of high amounts of intracellular substances and metabolites into the blood. Kidneys do not completely eliminate these substances, resulting in a range of serious metabolic disorders and clinical symptoms. This condition is known as TLS. Main clinical manifestations of TLS include hyperkalemia, hyperphosphatemia, hyperuricemia, and hypocalcemia. In severe cases, patients may develop acute renal failure and severe arrhythmia. Incidence of TLS in hematological malignancies is significantly higher compared with that of solid tumors, especially large volume tumors and tumors characterized by vigorous metabolism, such as B-cell lymphoma, which has the highest risk of TLS (103). Use of CAR-T cell therapy as an anti-tumor therapy may also cause TLS.

Principles of treatment of TLS are similar despite the cause. Prevention and treatment principles of TLS include (a) Adequate hydration (however, for elderly patients with chronic heart or kidney disease, attention should be paid to the input); (b) Urine alkalization is no longer recommended due poor efficacy and can also precipitate calcium phosphate in the renal tubules; (c) Hypouricemic agents can be used. For patients with low or moderate risk, allopurinol can be used as a first preventive drug (104, 105). Rasburicase is the preferred preventive drug for patients at high risk of TLS, and the preferred treatment drug for TLS patients (106). Rasburicase is a recombinant urate oxidase that can convert uric acid into highly soluble allantoin, and should not be administered in patients with glucose-6-phosphate dehydrogenase deficiency. (d) Diuretics are used to maintain urine volume, thus promoting the excretion of metabolites and potassium ions; (e) Correcting electrolyte disturbance. However, asymptomatic hypocalcemia should not be corrected, to prevent occurrence of nephrocalcinosis. (f) Use of continuous renal replacement therapy (CRRT).

### B-Cell Dysplasia

Currently, CD19 is the most frequently used target for CAR-T cells in treatment of hematological malignancies. Other targets include CD20, CD22, CD23, CD33, and CD123. CD19 is highly expressed on benign and most malignant B cells (non-B cells are characterized by low expression levels) (107). In addition to targeting tumor cells expressing target antigens, CAR-T cells attack normal B cells expressing target antigens, causing damage to normal B cells and ultimately leading to B-cell dysplasia (29, 99, 100, 108). B-cell dysplasia is common in CAR-T cell therapy targeting CD19 and is reported in all patients responding to treatment (100). B-cell dysplasia can last for a year (29) or even longer (4 years) (99) after disappearance of CAR-T cells in the body. The patient presents with hypogammaglobulinemia and is susceptible to infections (99). Treatment measures include gamma globulin infusion (99, 100) and prevention of infections.

### Hemophagocytic Lymphohistiocytosis (HLH)/Macrophage Activation Syndrome (MAS)

HLH is a clinical syndrome characterized by excessive inflammation. It is caused by abnormal proliferation of lymphocytes and tissue cells, resulting in release of high levels of inflammatory cytokines. The main clinical manifestations of HLH include fever, hepatosplenomegaly, abnormal liver function, decreased blood cells, increased triglycerides, increased serum ferritin [≥500μg/L (109)] and decreased fibrinogen levels (110). Studies on etiology report that HLH may be caused by congenital inheritance or secondary to autoimmunity and malignancy or infection (111). MAS is a secondary HLH (sHLH). MAS is a clinical syndrome caused by excessive activation and proliferation of T cells and macrophages, resulting in release of large quantities of inflammatory cytokines. Clinical manifestations of MAS are similar to those of HLH; however, it is characterized by high incidence of central nervous system symptoms and bleeding tendency, elevated serum ferritin [≥684μg/L (112)]. Notably, MAS may not be characterized by blood cell reduction (113).

HLH/MAS is a relatively rare disease with high mortality [up to about 80% (114, 115)] and a poor prognosis. Incidence of HLH/MAS in CAR-T cell therapy is approximately 3.48% (116). Clinical manifestations of HLH/MAS and CRS are similar; therefore, it is difficult to distinguish diagnosis of the two. Some studies report that HLH/MAS is a severe manifestation of CRS.

Mechanisms of CAR-T-related HLH/MAS include (a) Lysis of tumor cells result in release of large quantities of inflammatory cytokines and pro-inflammatory cytokines (22, 117); (b) Induction of CD8+ T-cells by pro-inflammatory factors results in production of high amounts of Th1 cytokines, such as IFN-γ, TNF-α, and IL-6, which forms a positive feedback loop of inflammation (117); (c) Activated CAR-T cells can release numerous cytokines (23, 24); (d) IFN-γ is correlated with MAS, high levels of IFN-γ are correlated with severe MAS (118). IFN-γ activates macrophages, and the activated macrophages release more inflammatory cytokines (22, 24–26); (e) Serum ferritin in these patients is significantly elevated (>10,000μg/L) (47), and ferritin itself is an inflammatory mediator. Furthermore, high levels of serum ferritin can promote release of inflammatory factors by activating NF-κB signaling pathway (119); (f) Some viral infections such as Epstein-Barr virus (*EBV*) induce occurrence of sHLH (120).

Currently, there is no targeted treatment approaches available for HLH/MAS patients. In principle, more aggressive immunosuppressive therapies should be given in early stages. Glucocorticoids are the main mode of treatment. This includes intravenous injection of methylprednisolone (1g/day, continuous 3-5 days), in combination with gamma globulin (1g/kg, continuous for 2 days), the regimen can be repeated on day 14 (121). If clinical deterioration occurs after treatment or existence of sHLH is confirmed, anakinra (IL-1 receptor antagonist) should be administered (115, 122). Further, etoposide is administered to refractory patients (115, 122). Etoposide (111) or rituximab (123) is given for sHLH caused by *EBV* infection. Cyclosporine, a second line drug causes neurotoxicity (124) and should be avoided for patients with central nervous system symptoms and can be substituted with anakinra (122). Moreover, combined medication can be used.

### Coagulation Disorders

Coagulation dysfunction often occurs during treatment with CAR-T cell therapy. Approximately 51%-56.6% of patients with hematological malignancies develop coagulation disorders after receiving CAR-T cell therapy (8, 125). Coagulation disorders occur within 6-20 days after infusion of CAR-T cells (125). Coagulation disorders associated with CAR-T cell therapy mainly include increased D-dimer, increased fibrinogen degradation products, prolonged prothrombin time, decreased fibrinogen, and thrombocytopenia. Further exacerbation of coagulation dysfunction can cause disseminated intravascular coagulation (DIC). Currently, only a few studies report on the incidence of DIC related to CAR-T cell therapy. A previous study reports about 7% incidence (125), whereas a different one reports that the incidence is about 28.3% (8). Notably, studies report that the incidence of coagulation disorders and DIC is higher in patients with severe CRS (8). In addition, the severity of coagulation disorders is positively correlated with the grade of CRS (125).

**Figure 3:**
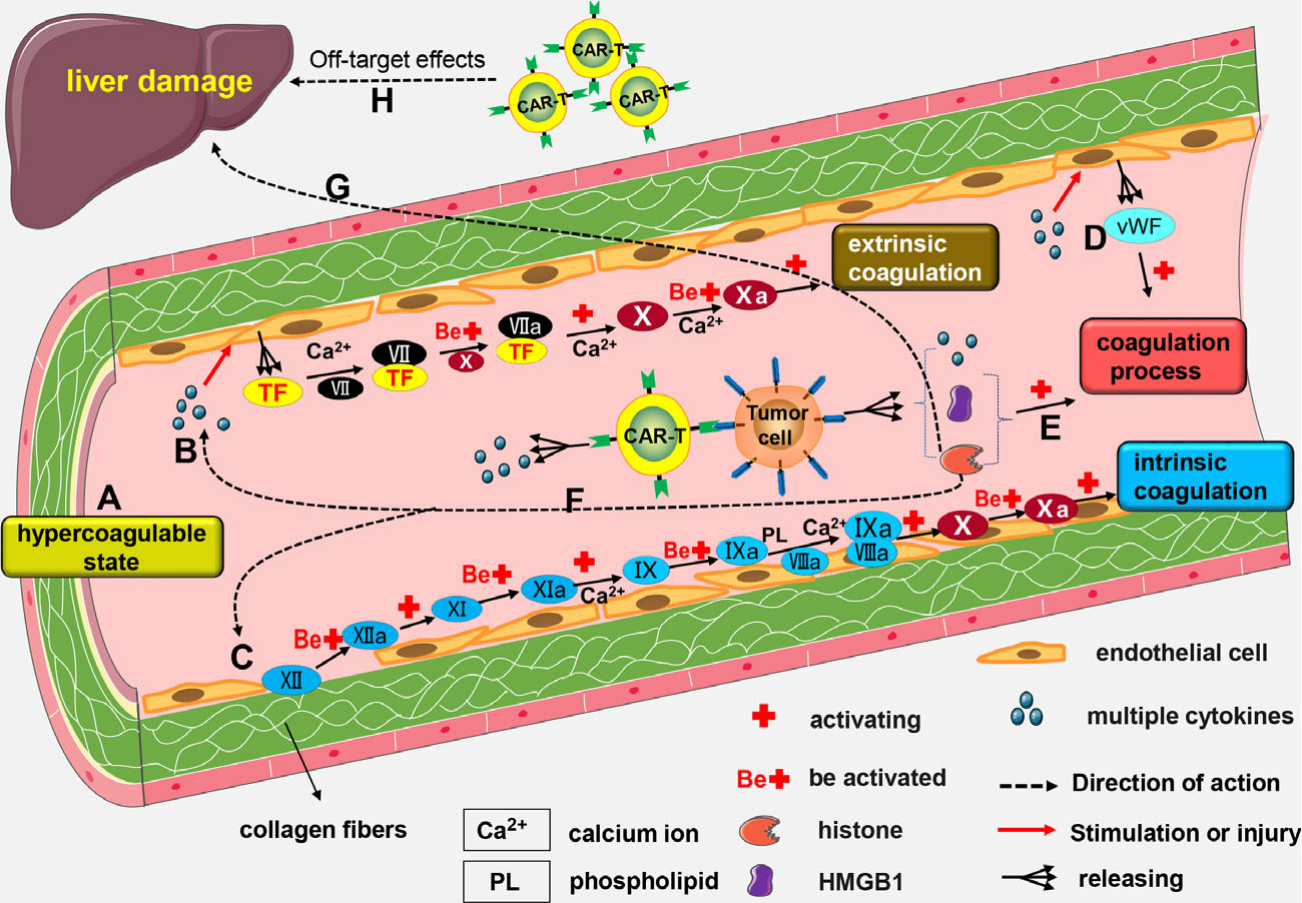
The mechanism of CAR-T-related coagulation dysfunction. **(A)** Blood from patients with malignant tumors is in a hypercoagulable state; **(B)** High number of cytokines can trigger the formation of lesions by vascular endothelial cells, thereby promoting the release of TF. Calcium (Ca2+) facilitates the combination of FVII with TF to form the FVII/TF complex. Activated FX enhances the activation of FVII/TF complex to form FVIIa/TF complex. Calcium (Ca2+) mediates the activation of FX by VIIa/TF complex to form FXa. This results in the activation of the extrinsic coagulation pathway. **(C)** Damage to endothelial cells exposes the underlying collagen fibers. Next, FXII combines with the exposed collagen fibers to form FXIIa. FXI is activated by FXIIa and transformed into FXIa. FIX is then activated by FXIa and transformed into FIXa; a process driven by Ca2+. FIXa and FVIIIa combine to form a IXa/VIIIa complex under the regulation of Ca2+ and PL. The IXa/VIIIa complex complex activates FX and transforms it into FXa leading to the activation of intrinsic coagulation pathway. **(D)** Activated endothelial cells release vWF factor which contributes to blood coagulation. **(E)** Rupture of some malignant tumor cells results in the release of HMGB1 and histones, both of which cause coagulation dysfunction. **(F)** Histones can indirectly activate the intrinsic or extrinsic coagulation pathway. **(G)** Excessive production of histones into the blood stream may cause liver damage and impair the production of coagulation factors. **(H)** Severe damage to the liver cells cause by the off-target effects of CAR-T cells may also impair the production of coagulation factors.

Mechanisms of CAR-T-related coagulation disorders are not fully known, and may be linked to the following mechanisms: (a) Blood from patients with malignant tumors is in a hypercoagulable state (126); (b) High levels of cytokines like IL-6 and TNF-α in the blood cause activation and lesions of vascular endothelial cells, resulting in increased release of tissue factor (TF) (127, 128). Coagulation factor VII (FVII) combines with TF to form FVII/TF complex. A series of reactions activates the extrinsic coagulation pathway; (c) Damage to endothelial cells affects their integrity, and collagen fibers below the endothelial cells are exposed. Subsequently, coagulation factor XII (FXII) combines with exposed collagen fibers and are activated to form FXIIa. Activation of several factors by FXIIa activate the intrinsic coagulation pathway; (d) When a patient has severe CRS, levels of cytokines in the body are significantly increased, and these cytokines induce activation of vascular endothelial cells (27, 28). Activated endothelial cells release von Willebrand factor (vWF) (27, 55), which promotes blood coagulation (129); (e) Both high-mobility group box-1 (HMGB1) and histones promote blood coagulation (130, 131). Certain malignant tumor cells, such as leukemia cells, release HMGB1 and histone H3 after rupture, which promote coagulation dysfunction or DIC (132); (f) Histones injure endothelial cell (133, 134) thus indirectly activating intrinsic or extrinsic coagulation pathway; (g) Excess histones in the blood can also cause liver damage (135, 136), and serious liver damage affects production of coagulation factors; (h) Severe damage of liver cells caused by off-target effects of CAR-T cells affects production of coagulation factors (**Figure 3**).

Patients with coagulation disorders can be treated with conventional treatment approaches. Most patients with coagulation disorders can recover without intervention (125). When CRS is under control and the levels of multiple cytokines decrease, the coagulation disorders recover gradually, and progression of DIC is effectively inhibited (8). However, once DIC occurs, timely and effective intervention and treatment must be administered.

### Cytopenias

Cytopenia is a common adverse reaction in CAR-T cell therapy which is characterized by neutropenia, thrombocytopenia and anemia. Its incidence is not consistent, which can be attributed to different types of diseases and treatment options. Studies from the last three years (**Table 3**), report that incidence of hemocytopenia is high in CAR-T cell therapy. Furthermore, studies have reported that cytopenia is the most prevalent among all adverse reactions reported with ≥ grade 3 (13, 14).

**Table 3:** Summary of the incidence of cytopenias associated with CAR-T cell therapy in patients with hematological malignant tumors (partial data).

Time	Disease	CAR-T Cell Therapy	Phase	Case	Age(years)	Any Grade			Gade 3/4			Trial registration
						Neutropenia	anemia	thrombocytopenia	Neutropenia	anemia	thrombocytopenia	
2019	relapsed / refractory MM	BCMA-CAR-T cells	I	33	37~75	28 (85%)	19 (58%)	19 (58%)	28 (85%)	15 (45%)	15 (45%)	NCT02658929 (4)
2019	relapsed/ refractory B-ALL	CD19-CAR-T cells	I	25	1~22.5	3 (12%)	**---**	4 (16%)	3 (12%)	**---**	4 (16%)	NCT01860937 (5)
2019	relapsed / refractory MM	BCMA-CAR-T cells	I	25	44~75	**---**	**---**	**---**	11 (44%)	5 (20%)	7 (28%)	NCT02546167 (6)
2019	relapsed / refractory MM	BCMA-CAR-T cells CD19-CAR-T cells	II	21	18~69	20 (95%)	20 (95%)	16 (76%)	18 (85%)	13 (62%)	13 (62%)	ChiCTR-OIC-17011272 (9)
2019	relapsed or refractory diffuse large B-cell lymphomas	CD19-CAR-T cells	IIa	111	22~76	22 (20%)	53 (48%)	14 (13%)	22 (20%)	43 (39%)	13 (12%)	NCT02445248 (10)
2020	relapsed/ refractory B-ALL	CD19-CAR-T cells	I	23	10~67	**---**	**---**	**---**	17 (74.9%)	7 (30.4%)	9 (39.1%)	ChiCTR-ONN-16009862, ChiCTR-1800019622 (11)
2020	relapsed or refractory large B-cell lymphomas	CD19-CAR-T cells	I	269	54~70	169 (63%)	129 (48%)	84 (31%)	161 (60%)	101 (37%)	72 (27%)	NCT02631044 (12)
2020	relapsed / refractory mantle-cell lymphoma	CD19-CAR-T cells	II	68	38~79	59 (87%)	46 (68%)	50 (74%)	58 (85%)	34 (50%)	35 (51%)	NCT02601313 (13)
2021	relapsed / refractory MM	BCMA-CAR-T cells	II	128	33~78	117 (91%)	89 (70%)	81 (63%)	114 (89%)	77 (60%)	67 (52%)	NCT03361748 (14)

MM, multiple myeloma; B-ALL, B-cell acute lymphoblastic leukemia; BCMA, B-cell maturation antigen.

—: Relevant data are not mentioned in the experiment.

To improve the efficacy of CAR-T cells, patients should be given the lymphodepleting chemotherapy regimen before CAR-T cell therapy. Currently, the most commonly used regimen is the combination of fludarabine and cyclophosphamide. Patients often develop cytopenias after receiving lymphocyte clearance therapy. Early cytopenias may be as a result of lymphatic failure chemotherapy (137).

It has been shown that cytopenias developing after CAR-T cell therapy may last for a long time, exceeding 30 days (10, 13, 15, 138, 139). This phenomenon is termed as prolonged hematologic toxicity (PHT), and it is characterized with ≥ grade 3 neutropenia or thrombocytopenia following CAR-T cells infusion(exceeding 30 days) (15). In a phase I clinical trial that tested the efficacy of CD19-CAR-T cells in the treatment of patients with relapsed/refractory diffuse large B-cell lymphoma (R/R DLBCL), it was found that 18 of 31 (58%) patients developed PHT (15). Moreover, the 1-year overall survival (OS) (36%) of patients with PHT was significantly lower than the 1-year OS (81%) of patients without PHT.

Although the development of PHT is not well understood, it may be triggered by the following factors: (a) PHT can be caused by a previous administration of higher-intensity chemotherapy (137); which may deteriorate hematopoietic function, and hence decrease the production of blood cells in the long run.(b)Patients with a history of hematopoietic stem cell transplantation (HSCT) and CRS are likely to develop PHT (137, 140). Notably, patients with high-grade CRS may have severe cytopenias and require longer recovery time compared to those with low-grade CRS (27). Usually, patients are treated with HSCT, followed by CAR-T cell therapy. The interval between these two treatments usually exceeds 1 year. HSCT can cause damage to the patient’s hematopoietic function. The impaired hematopoietic function may not have fully recovered, and then the patient suffers a second blow (receiving CAR-T cell therapy). This mode of treatment may cause or even worsen PHT.

The treatment of cytopenia: (a) Patients with early and mild cytopenias, symptomatic and nutritional support are recommended. For such patients with neutropenia, active prevention or anti-infective treatments can also be given. (b) For patients with long-term neutropenia, granulocyte-colony stimulating factor (G-CSF) treatment is recommended. Currently, the U.S. Food and Drug Administration has approved the use of G-CSF (filgrastim) in the treatment of congenital and acquired neutropenia (141). The recommended dose of G-CSF is 5 mcg/kg/day (141). Other dosage forms of G-CSF include pegfilgrastim and lenograstim. (c) Patients with long-term neutropenia and thrombocytopenia may benefit from GM-CSF treatment at a dose of 250 mcg/m2/day (141), but it should not be administered in the first 3 weeks after injection of KYMRIAH (a CD19-CAR-T cell therapy) or before CRS is resolved (142). The dosage forms of GM-CSF include sargramostim and molgramostim. (d) For patients with long-term and severe anemia and thrombocytopenia, red blood cell and platelet transfusion is recommended (143). (e) In cases of severe and prolonged cytopenias, eltrombopag (Thrombopoietin-receptor agonists, 50-150mg/day) has been used previously (15). This dose produced a median time for blood system recovery of 123 days (range: 41 to 145 days) in four patients. (f) Autologous or allogeneic stem cell transplantation is proposed as a potential treatment for cytopenia (140, 144), but the efficacy of this treatment has not been clarified through clinical trials.

## Future Prospects of CAR-T Cell Therapy

As an emerging anti-tumor therapy, CAR-T cell therapy has made great achievements in the treatment of hematological malignancies. Currently, this therapy has begun to be applied to the research and treatment of solid tumors. However, it is associated with adverse reactions which limits its clinical application to a certain extent. Only by minimizing the incidence and impact of these adverse reactions can the safety of CAR-T cell therapy be effectively enhanced. By effectively preventing and treating these adverse reactions, more and more tumor patients will benefit from CAR-T cell therapy.

## Author Contributions

LM: Writing-Original draft preparation, Investigation, table and figure preparation. ZZ: Investigation, table and figure preparation. ZR: Investigation. YL: Conceptualization, Methodology, Supervision. All authors contributed to the article and approved the submitted version.

## Funding

This work was supported by Special Research Project of Lanzhou University Serving the Economic and Social Development of Gansu Province (054000282), Lanzhou Talent Innovation and Entrepreneurship Project (2020-RC-38), and Supported by the Fundamental Research Funds for the Central Universities (lzujbky-2020-kb14).

## Conflict of Interest

The authors declare that the research was conducted in the absence of any commercial or financial relationships that could be construed as a potential conflict of interest.
